# The change in energy absorbed post removal of metalwork in a simulated paediatric long bone fracture

**DOI:** 10.1007/s11832-014-0606-z

**Published:** 2014-08-27

**Authors:** Alan J. Howieson, Michael D. Jones, Peter S. Theobald

**Affiliations:** Trauma Biomechanics Research Group, Institute of Medical Engineering and Medical Physics, Cardiff University, Queen’s Building, The Parade, Cardiff, CF24 3AA UK

**Keywords:** Diaphyseal fracture, Paediatric, Open reduction internal fixation, Elastic stable intramedullary nailing

## Abstract

**Purpose:**

The surgical treatment of paediatric fractures is increasing. Open reduction and internal fixation (ORIF) with plates and screws is long established, whilst the use of elastic stable intramedullary nailing (ESIN) has become increasingly popular. This study quantifies, in terms of the energy required to produce a fracture, the biomechanical sequelae of both techniques post removal of metalwork, to provide clinicians with evidence to guide post-operative advice.

**Methods:**

An immature bovine model was adopted to ascertain whether these techniques exposed the bone to a greater re-fracture risk following removal of the device. Bones were prepared to reflect ORIF or ESIN techniques, or prepared intact for the acquisition of control data. Each bone was tested to failure at 90 °/s, with the absorbed energy then being calculated to determine the relative difference between each technique and versus control data. Data describing peak shear stress and torque were recorded.

**Results:**

Absorbed energy was reduced by 47 % in the ORIF group compared to both the control (*p* = 0.011) and ESIN (*p* = 0.018) groups. The peak shear stress and torque were also significantly different. All ORIF bones failed through drill holes, suggesting stress localisation around the defects.

**Conclusion:**

This study suggests that there is a significantly higher re-fracture risk following the removal of ORIF plates when compared to both ESIN and the control environment. Whilst this may reflect the intuitive view of many clinicians, this study provides a quantitative value of the reduction in strength and should help clinicians to appropriately caution patients and parents prior to surgery.

## Introduction

Historically, the vast majority of paediatric fractures have been managed non operatively, but a ten-fold increase in operative treatment rates for paediatric forearm fractures was reported over the last two decades of the 20th century [1, 2]. Open reduction and internal fixation (ORIF) with plates and screws was the traditional surgical technique, although elastic stable intramedullary nailing (ESIN) has become increasingly popular [3–9]. The need to remove both ORIF and ESIN implants from children following bone healing is, however, associated with a relatively high risk of re-fracture [10–12].

The removal of ORIF implants exposes a series of cortical screw holes, which gradually heal over time. Intuitively, ESIN would, therefore, be associated with a lower risk of re-fracture following implant removal, but this risk has never been quantified and is extremely hard to define experimentally. A reduction in energy absorbed before fracture is one quantifiable variable that would give some indication as to whether there is a difference between the techniques and, to date, there are no published studies examining this factor.

There are many reasons for the removal of metalwork in children: all metal implants, with the exception of titanium, will lead to corrosion over time and the release of metal ions into the body. Whilst tumour development next to metal implants has been reported, reports are limited to case reports and large-scale studies are difficult to implement. There is also the risk of foreign body reactions and infection. Pain from prominent implants, implant migration, implant failure, wound problems and allergy are other problems [10]. The potential short- and long-term problems are illustrated by two case reports both involving children who had their femur plated at the age of 6 years: one child sustained a re-fracture just distal to the plate at the age of 12 years and the other sustained a re-fracture at the age of 67 years—radiographs taken at the time of re-fracture showed almost complete loss of bone under the plate due to stress shielding [10].

Experimental evidence regarding the force required to fracture a paediatric long bone is scare and no relevant published studies were identified which quantified its magnitude. Forearm fractures commonly result in a spiral pattern or with the fracture lines at different levels on the radius and ulna, which suggests a torsional force [13]. The energy involved in falls from a height can be calculated using Newton’s equations of motion. Calculations necessarily include complex equations which include a tip mechanism, an estimation of the body’s centre of gravity and joint movements during the fall, and changes in linear momentum: joint movement in particular makes calculating ground reaction forces extremely difficult [14].

This study focuses on comparing the biomechanical consequences of the operative treatment of paediatric long bone fractures with either ORIF or ESIN, with the aim of providing clinicians with a quantitative difference between the two techniques and between intact normal bone. Immature bovine metacarpals were chosen as a representative model, having similar dimensions to paediatric femora. The primary outcome measure was the reduction in energy absorbed between the two techniques and versus normal bone. Secondary outcome measures were the maximum torque to failure, peak shear stress, shear modulus and fracture pattern.

## Materials and methods

Bovine metacarpals, which have similar dimensions to human paediatric femora, were used as a model to explore the reduction in energy absorption and, thus, identify the fixation technique most likely to cause re-fracture. Metacarpal bones (*n* = 18) were harvested following normal slaughter of commercial cows (<1 year old), before being cleared of all soft tissues and inspected for any previous fractures or obvious defects that could potentially affect the results. The diaphyseal lengths and central diameters were recorded (mean value of three measurements), before identifying the centre of rotation through the longest axis. The bones were then randomly sub-divided to form equal groups of control, ORIF and ESIN specimens.

**Fig. 1:**
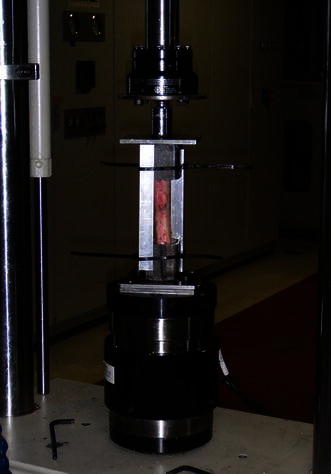
The alignment phase of the experimental preparations necessitated the use of two right-angled supports to achieve alignment of the distal and proximal bone ends prior to securing with Wood’s metal. Aligning the centroidal axis of the bone to the vertical ensured a consistent application of load across all samples

ORIF specimens were prepared by using a broad dynamic compression plate as a template. Three bi-cortical holes were drilled (2.5 mm diameter) and tapped (3.5 mm diameter), using the template, on either side of the bone mid-point. This represented the recent removal of a plate fixed with six, 3.5-mm bicortical screws. The cortical defect ratio, a measure of the extent of the defect caused by the screw-hole, was calculated for all samples. The ESIN specimens were prepared by identifying the centre of the proximal joint and by passing a wire (5.6 mm diameter) from this point along the length of the intramedullary canal, before removing it and, thus, leaving an intramedullary defect. The wire diameter was specifically calculated and chosen to represented the insertion of two flexible intramedullary nails, each with a diameter 30–40 % of the internal diameter of the bone, as per standard practice. Each bone was then embedded to a depth of 50 mm in Wood’s alloy (Flinn Scientific, Inc., USA), before being clamped into a tension–torsion testing machine (MTS 858 Mini Bionix II) (Fig. 1).

Each bone was tested in torsion to failure, at a rotational rate equal to 90 °/s. This rate was adopted from a previous study, in view of the absence of literature quantifying the rotational rate experienced during equivalent, ‘real-life’ fracture scenarios [15]. Previous biomechanical studies have shown that testing in torsion is the most valid method for assessing the energy absorbed to failure [16]. Data (time, torsional load) were automatically acquired at 100 Hz, via a wired personal computer, whilst the cortical thickness was retrospectively measured at the previously identified mid-shaft position. All data were then analysed to calculate the shear stress (Eq. 1), shear modulus (Eq. 2) and energy absorption (Eq. 3):

1 $$\text{Shear}\;\text{stress}\;=\;\frac{\text{bone}\;\text{radius}\;\times \;\text{torque}}{\text{polar}\;\text{moment}\;\text{of}\;\text{inertia}}$$

2 $$\text{Shear}\;\text{modulus}\;=\;\frac{\text{bone}\;\text{length}\;\times \;\text{torque}}{\text{polar}\;\text{moment}\;\text{of}\;\text{inertia}\;\times \;\text{rotated angle}}$$

3 $$\text{Energy}\;\text{absorbed}\;=\;\frac{\text{rotated angle}\;\times \;\text{torque}}{2}$$

MS Excel 2010 (Microsoft Corporation, USA) and GraphPad InStat version 3 (Graphpad Software, Inc., USA) were used to compute analysis of variance (ANOVA) and Student’s *t*-test, enabling the identification of any statistical significance between the datasets. Data were considered statistically significant when *p* < 0.05.

## Results

**Fig. 2:**
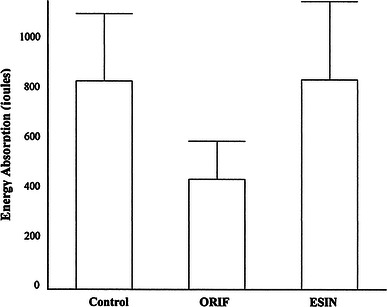
Data describing the energy absorption across all tests

Data describing the dimensions of the immature bovine bones and the performance of the bones, when subject to a torsional load-to-failure, are presented in Table 1 and Fig. 2, respectively. There were no significant differences between the dimensions of the three groups. A 47 % reduction in energy absorption was observed by the comparison of ORIF to both the ESIN (*p* = 0.018) and control (*p* = 0.011) datasets; however, there was no significant difference between the ESIN and control datasets (Fig. 2). All ORIF bones had a cortical defect ratio 0.12–0.16, with all failing through at least one drill hole.

**Table 1:** Data describing the mean dimensions of each group

	Mean length (mm) ± SD	Mean polar moment of inertia (×10^−9 ^m^4^) ± SD	Mean cortical thickness (mm) ± SD
Control	204 ± 8	18.9 ± 1.99	4.4 ± 0.7
ORIF	207 ± 12	18.5 ± 2.14	5.0 ± 0.6
ESIN	194 ± 9	16.7 ± 5.23	4.9 ± 0.6

Table 2 describes the secondary outcome measures. ORIF specimens required significantly less torque to produce fracture than the control dataset (*p* = 0.049); ESIN bones also required less mean torque to produce fracture than the control, but the results were not significant (*p* = 0.507). Direct comparison of the ESIN and ORIF specimens revealed that the former required a higher torque to produce fracture, although the difference between the two did not reach statistical significance (*p* = 0.247). The maximum shear stress differed significantly when comparing the control and ORIF data (*p* = 0.043).

**Table 2:** Data describing the mean biomechanical values of each group

	Mean angular deformation to failure (°)	Mean peak torque (Nm)	Mean energy absorbed (joules)	Mean polar moment of inertia (×10^−9^ m^4^)	Mean maximum shear stress (MPa)
Control	23.2	70.6	835	18.93	40.3
ORIF	15.9	54.5	443	18.50	31.3
ESIN	26	64.1	843	16.59	39.8

The shear modulus for immature bovine bone was found to be in the range 15.5–18.8 GPa and provides a reference value for further studies.

## Discussion

This study helps to quantify the reduction in bone strength following the removal of paediatric internal fixation. There are numerous clinical reports of long bone re-fractures: Kanlic et al. [17] reported a case after plate removal from the femur and, in a different multicentre study, Flynn et al. [18] reported re-fracture after nail removal. Re-fracture after paediatric forearm fracture occurs more commonly than in any other bone [12]. Multiple cases have been published: Deluca et al. [19] reported seven re-fractures from a set of 37 adults who had plates removed from the forearm: four of these patients required further open surgical treatment and three were treated with a cast. All arms were protected for a period of 4–8 weeks post removal of metalwork, although the metalwork was removed at an average of 87 days. Nielsen and Simonsen [20] removed plates at a mean period of 26 weeks post surgery in 23 children with fractures of the forearm, and one child sustained a re-fracture 3 months after the plate was removed. Vainionpää et al. [21], as part of a large series of 192 children with fractured forearms, reported one re-fracture amongst a series of ten patients treated with ORIF; the plates were removed at a range of 6–14 months post injury. Kim et al. [11] examined specific complications related to the removal of metalwork in paediatric forearm fractures and found, from a total of 43 children with a mean age of 10.6 years, that there were three re-fractures, two of which occurred in the same patient.

The clinical relevance of the bone model adopted in this study is confirmed by considering the favourable comparison with pre-pubertal female and male femora. The average overall diameter for all the bovine bones tested was 21.2 mm, with an average cortical thickness of 4.8 mm. Previously published femoral dimensions from children with an age range of 8.2–9.4 years found an average femora diameter in pre-pubertal females and males of 19.9 and 21.0 mm, respectively, with cortical thicknesses of 4.6 and 5.0 mm [22–24].

It has also been shown by Vashishth et al. [25] that, whilst the elastic modulus of bovine bone is different from human bone, the ratio of yield stress in torsion, compression and tension correlates well with human bone and they have been used in previous studies to model human bone [26]. We calculated the mean shear modulus for our immature bovine bone specimens to be in the range 15.5–18.8 GPa—this provides a useful reference value for further work.

The diameter of the ESIN wire used corresponds to the insertion of two flexible intramedullary nails measuring 30–40 % of the internal diameter of the bone, as recommended for standard practice. Unfortunately, radiographic facilities were not available to examine the relative position of the wire within the canal; however, this potential variation was deemed unlikely to have any significant influence on the data. It was also important that the size of instruments used in this study reflected clinical practice to allow some extrapolation of results.

A significant difference in energy absorbed to failure was observed between the ORIF and control groups, data which correlate to that of Edgerton et al. [27], who report a 20 % reduction in strength for a single drill hole using sheep femora. Our data, which describe multiple drill holes, found a greater, 47 %, reduction in absorption; however, compared to the data of Brooks et al. [28], our reduction in strength with multiple drill holes is less than the 55 % reduction they noted for a single hole in canine bone. It should be noted, however, that our specimens had a cortical defect ratio of 012–0.16, whereas Brooks et al. report a ratio of 0.14–0.18 [28]. This correlates with finite element simulations, as comparing the cortical defect ratio reported here with other studies, simulations have predicted little decline in torsional strength up to a defect ratio equal to 0.1, then a rapid decline in torsional strength between ratios of 0.1–0.2, before declining less steeply above 0.2 [27, 29]. Furthermore, the qualitative data acquired during this investigation clearly indicates that drill holes do localise stress, with all ORIF bones failing through at least one such cortical defect.

The effect of occluding a drill hole was reported by Ho et al. [30]. They found that a 4-mm-diameter bicortical defect led to a 38 % reduction in energy absorbed by healthy porcine femora but that, by occluding the hole with plaster of Paris, there was a negligible difference between the specimen and intact bone.

In conclusion, this study has adopted an appropriately dimensioned, accepted animal model to evaluate the change in energy absorbed post simulated removal of a fixation device in pre-pubertal children. The data presented suggest that children are at a higher risk of re-fracture immediately following ORIF removal than at an equivalent phase post ESIN. It would be interesting to know how the strength of the bone recovered over time as the defect consolidated, but this study would be impossible to perform in the paediatric population and very difficult even using animal models. For the purpose of this study, we have isolated one important variable, namely the cortical defect: future areas of interest include the effect of disuse osteopaenia and the relevance of the entry point of the nail in the metaphyseal region of the bone.
